# Die Rolle von Psychotherapeut:innen bei Anfragen nach assistiertem Suizid – eine ungeklärte Frage

**DOI:** 10.1007/s00103-026-04201-1

**Published:** 2026-02-10

**Authors:** Pola Hahlweg

**Affiliations:** Institut und Poliklinik für Medizinische Psychologie, Universitätsklinikum Hamburg-Eppendorf, Martinistr. 52, 20246 Hamburg, Deutschland

**Keywords:** Todeswünsche, Sterbehilfe, Klinische Psychologie, Kompetenzen, Narrative Übersicht, Wishes for hastened death, Aid in dying, Clinical Psychology, Competencies, Narrative review

## Abstract

Anfragen von Patient:innen nach assistiertem Suizid sind ein komplexer, longitudinaler und iterativer Prozess. Für Gesundheitsfachpersonen ergeben sich in diesem Prozess potenziell verschiedene kommunikative und evaluative Aufgaben, wie das Informieren über Handlungsalternativen, das Erfassen psychischer Belastung und ggf. Diagnostizieren psychischer Störungen und die Prüfung der Freiverantwortlichkeit. Aufgrund einer ausstehenden gesetzlichen Regelung in Deutschland bestehen aktuell keine dezidierten Pflichten für Psychotherapeut:innen bei assistiertem Suizid. Diese narrative Übersichtsarbeit spezifiziert zunächst allgemeine Aufgaben bei Anfragen nach assistiertem Suizid und überträgt diese anschließend auf Psychotherapeut:innen. Dabei wird differenziert zwischen rechtlichen und standesrechtlichen Pflichten („müssen“/„nicht dürfen“), fachlichen Kompetenzen („können“) und daraus abgeleiteten normativen Empfehlungen („sollten“).

Die standesrechtlichen Vorgaben und psychotherapeutischen Kompetenzen verdeutlichen, dass Psychotherapeut:innen qualifiziert sind, um sich an Anfragen nach assistiertem Suizid zu beteiligen. Gleichzeitig sollten die möglichen Konsequenzen für die psychotherapeutische Rolle, die sich aus einer Beteiligung an einer Anfrage mit dem Ziel der Lebensbeendigung ergeben, gründlich reflektiert und geeignete Kommunikationsstrategien entwickelt werden. Zudem ist auf eine klare Abgrenzung zwischen unterstützenden und evaluativen Tätigkeiten zu achten. Die Freiwilligkeit der psychotherapeutischen Beteiligung muss gewahrt bleiben, insbesondere das Recht auf Nichtbeteiligung aus Gewissensgründen. Insgesamt scheint der potenzielle Nutzen einer Beteiligung von Psychotherapeut:innen an Anfragen nach assistiertem Suizid die möglichen Nachteile deutlich zu überwiegen.

## Abstract (en)

Patients’ requests for assisted suicide involve a complex, longitudinal, and iterative process. For healthcare professionals, this process potentially involves various communicative and evaluative tasks, such as providing information about alternative courses of action, assessing psychological distress and, if necessary, diagnosing mental disorders and examining free decision-making capacity. Due to pending legislation in Germany, there are currently no specific obligations for psychotherapists regarding assisted suicide. This narrative review first specifies general tasks in requests for assisted suicide and then applies these to psychotherapists. A distinction is made between legal and professional obligations (“must”/“cannot”), professional competencies (“can”), and normative recommendations derived from these (“should”).

Psychotherapists’ professional code of conduct and competencies show that they are qualified to participate in requests for assisted suicide. At the same time, possible consequences for the psychotherapeutic role resulting from participation in a request that aims to end one’s life should be thoroughly considered and appropriate communication strategies developed. In addition, a clear distinction must be made between supportive and evaluative activities. The voluntary nature of psychotherapists’ involvement must be preserved, in particular the right to conscientious objection. Overall, the potential benefits of psychotherapists’ involvement in requests for assisted suicide appear to clearly outweigh the possible disadvantages.

## Einleitung

Um die (potenzielle) Rolle von Psychotherapeut:innen bei Anfragen von Patient:innen nach assistiertem Suizid einordnen zu können, ist es wichtig, sich der Komplexität und Kontextabhängigkeit des Themas bewusst zu sein. Zum assistierten Suizid gehört nicht nur die konkrete zum Tode führende Handlung, sondern auch eine oft lange und nicht immer geradlinige Auseinandersetzung mit dem Thema, die sich grob in folgende Phasen gliedern lässt: Annäherung, Beurteilung, Vorbereitung, Durchführung und Nachsorge [1]. Diese Phasen können sich überlappen und bestehen aus informellen und formalen Anteilen. In den Prozess können neben der sterbewilligen Person auch deren Angehörige, verschiedene Ärzt:innen, weitere Gesundheitsfachpersonen und Mitarbeitende von Sterbehilfevereinen involviert sein [1]. Der Prozess findet intra- und interindividuell statt und kann von Widersprüchen, Konflikten, Ambivalenz oder sich verändernden Standpunkten geprägt sein. Dies macht deutlich, dass es sich bei assistiertem Suizid um einen hochkomplexen und an vielen Stellen psychisch-emotionalen sowie sozial-interaktionellen Prozess handelt.

Zudem ist der assistierte Suizid kontextabhängig, beispielsweise von der jeweiligen gesetzlichen Regelung. In Deutschland schränkte der von 2015 bis 2020 bestehende § 217 im Strafgesetzbuch (StGB) auch Suizidassistenz durch Ärzt:innen und weitere Heilberufe ein [2]. Seit der Abschaffung dieses Paragraphen durch das Bundesverfassungsgerichtsurteil im Februar 2020 ist dies nicht mehr der Fall [3]. Gleichzeitig betonte das Bundesverfassungsgericht, dass niemand zur Suizidassistenz verpflichtet werden kann, es also ein Recht auf Ablehnung der Beteiligung aus Gewissensgründen gibt. Im Urteil wurde die Freiverantwortlichkeit der sterbewilligen Person als die zentrale Voraussetzung für einen assistierten Suizid betont und 4 Kriterien dafür benannt: 1) freie Willensbildung, 2) umfassende Informiertheit, 3) Freiheit von unzulässiger Einflussnahme oder Druck und 4) Dauerhaftigkeit und innere Festigkeit. Eine neue gesetzliche Regelung und somit die genaue Ausgestaltung der Kriterien, Beteiligten, Strukturen und Abläufe stehen jedoch aus [4].

Aktuell ist in Deutschland noch eine offene Frage, in welchem Ausmaß Suizidassistenz innerhalb oder außerhalb des Gesundheitssystems stattfinden soll. Innerhalb des Gesundheitssystems werden unter den Heil- und Pflegeberufen traditionell vor allem Ärzt:innen als relevante Berufsgruppe für Suizidassistenz in den Blick genommen. Dies wird unter anderem in den abgelehnten Gesetzesentwürfen zur Neuregelung des assistierten Suizids in Deutschland aus den Jahren 2022/2023 deutlich [5–8]. Gleichzeitig zeigt sich beispielsweise in Kanada und den USA eine Ausweitung auf weitere Berufsgruppen wie Pflegefachkräfte [9–11]. Auch waren in einem der abgelehnten deutschen Gesetzesentwürfe Fachärzt:innen für Psychiatrie und Psychotherapie und Psychotherapeut:innen gleichermaßen für die Beurteilung der Freiverantwortlichkeit und der Zumutbarkeit eines zweiten Untersuchungstermins vorgesehen [8]. Zunehmend wird das Potenzial gesehen, das im Einbezug weiterer Heil- und Pflegeberufe liegt. Allerdings ist dieser bisher begrenzt und unzureichend ausgestaltet [1].

Deshalb beschäftigt sich dieser Übersichtsbeitrag mit der (potenziellen) Rolle von Psychotherapeut:innen bei Anfragen nach assistiertem Suizid in Deutschland. Dazu wurde eine Literaturrecherche in PubMed durchgeführt (siehe Onlinematerial), wobei auch internationale Literatur einbezogen wurde. Die Autorin ist psychologische Psychotherapeutin mit Schwerpunkt auf Verhaltenstherapie und Psychoonkologie und hat langjährige Erfahrung in der Forschung zu patient:innenzentrierter Gesundheitsversorgung und assistiertem Suizid. In diesem Artikel werden zunächst generelle Aufgaben bei Anfragen nach assistiertem Suizid spezifiziert. Im Anschluss werden diese auf Psychotherapeut:innen bezogen. Dabei wird in den Blick genommen, was Psychotherapeut:innen *müssen* und *(nicht) dürfen* (im Sinne (standes-)rechtlicher Pflichten), was sie *können* (im Sinne einschlägiger Expertise und Kompetenzen) und was sie folglich *sollten* (im Sinne einer normativen Empfehlung).

## Aufgaben bei Anfragen nach assistiertem Suizid

Aufgrund der Vielschichtigkeit und Prozesshaftigkeit von Anfragen nach assistiertem Suizid ergeben sich im Verlauf verschiedene Rollen und Aufgaben. Dies gilt für die sterbewillige Person selbst, für ihre Angehörigen und für die beteiligten Gesundheitsfachpersonen. Um einen Überblick über die Aufgaben der Gesundheitsfachpersonen zu geben, werden im Folgenden hauptsächlich zwei qualitative Studien aus Kanada herangezogen: Crumley et al. befragten 2021 verschiedene Gesundheitsfachpersonen (Ärzt:innen, „nurse practitioners“, Pflegefachpersonen, Apotheker:innen, „health administrators“), die an assistierten Suiziden beteiligt sind, welche Rollen und Aktivitäten sie bei Anfragen nach assistiertem Suizid übernehmen, und beschrieben diese in einem umfangreichen Flussdiagramm [12]. Oczkowski et al. fassten 2021 die Abläufe und Aufgaben für Gesundheitsfachpersonen aus Sicht von sterbewilligen Personen, ihren Angehörigen und den Gesundheitsfachpersonen (Ärzt:innen, Pflegefachpersonen, Sozialarbeiter:innen, „managers“, die direkt am jeweiligen Prozess für assistierten Suizid beteiligt waren) zusammen [13]. Diese beiden Prozessdarstellungen wurden gewählt, da empirische Daten eingeflossen sind, sie einen hohen Detailgrad aufweisen und sich nicht spezifisch auf ein stationäres Setting beziehen. Zudem ist assistierter Suizid in Kanada im Gesundheitssystem verankert [14], was die Ableitung von Aufgaben für Gesundheitsfachpersonen erleichtert.

Eine relevante Gemeinsamkeit zwischen Kanada und Deutschland besteht darin, dass assistierter Suizid auf dem Recht auf Autonomie beruht und auch Personen offensteht, die keine unmittelbar zum Tode führende Grunderkrankung haben [3, 15]. Zugleich ist zu berücksichtigen, dass in Kanada – anders als in Deutschland – neben dem „assistierten Suizid“, bei dem die letzte Handlung durch die sterbende Person selbst ausgeführt werden muss, auch „Tötung auf Verlangen“ erlaubt ist, bei der die tödliche Substanz verabreicht wird. In Kanada erfolgt sogar fast ausschließlich Tötung auf Verlangen, mit einer der höchsten Zuwachsraten über die letzten Jahre [14, 15].

Vor diesem Hintergrund wurden aus den beiden Prozessdarstellungen [12, 13] diejenigen Rollen und Aufgaben abgeleitet, die für den deutschen Kontext relevant erscheinen. Aspekte, die aufgrund der benannten Unterschiede nicht übertragbar waren, wurden nicht berücksichtigt. Ziel der Zusammenstellung ist es, einen Überblick über (potenzielle) Aufgaben für Gesundheitsfachpersonen im Rahmen des assistierten Suizids in Orientierung an den Prozessphasen zu geben. Angesichts der Komplexität der Prozesse kann dabei kein Anspruch auf Vollständigkeit erhoben werden.

### Querschnittsaufgaben.

Oczkowski et al. benennen übergeordnet zwei Querschnittsaufgaben: einerseits die Koordination des assistierten Suizids, inklusive kontinuierlicher Unterstützung, Beratung und Erwartungsmanagement, andererseits personenzentrierte Versorgung, welche Würde und Privatsphäre sicherstellt, nicht stigmatisiert, vorherige Erfahrungen mit Tod und Sterben in den Blick nimmt und auch das private Umfeld der sterbewilligen Person unterstützt [13]. Auch andere Publikationen betonen die Notwendigkeit eines respektvollen und offenen Umgangs mit den sterbewilligen Personen und des Versuchs, deren Leid zu verstehen und bei der Sinnfindung zu unterstützen [16]. Kommunikation, Informieren und Beraten sind zentrale Aufgaben [10]. Zudem wird als übergeordnete Aufgabe benannt, im Gesundheitssystem und in der Gesellschaft ein Bewusstsein über assistierte Suizide zu schaffen [13]. Ein kontroverses Thema ist die Frage, ob die Initiierung eines Gesprächs über assistierten Suizid immer von der sterbewilligen Person ausgehen soll oder auch Gesundheitsfachpersonen assistierten Suizid von sich aus ansprechen dürfen oder sollen [17].

### Annäherungs- und Abwägungsphase.

In der Annäherungs- und Abwägungsphase ergeben sich primär Aufgaben der Klärung und der Informationsweitergabe [12, 13]. Im Verlauf ist – im Sinne der umfassenden Informiertheit – das Informieren der Person über ihre Erkrankungssituation sowie mögliche Alternativen zu einem assistierten Suizid essenziell [18]. Eine weitere Aufgabe kann sein, an andere Behandelnde zu verweisen [12]. Dies kann beispielsweise relevant sein, wenn eine spezialisierte Beratung z. B. zu einer somatischen Erkrankung nötig ist oder sich die angesprochene Gesundheitsfachperson aus eigenen Gewissensgründen nicht in der Lage sieht, ein Gespräch über assistierten Suizid zu führen. Für Deutschland hat die angemeldete S2k-Leitlinie „Umgang mit Anfragen nach Assistenz bei der Selbsttötung“ das Potenzial, national klare Handlungsempfehlungen zur Aufklärung und Beratung zu geben [19].

### Beurteilung.

Für die Beurteilung der Zugangsvoraussetzungen für assistierten Suizid sehen die meisten internationalen gesetzlichen Vorgaben zwei Prüfende vor [20]. Die Prüfenden beurteilen die Freiverantwortlichkeit und ggf. vorliegende psychische Erkrankungen [21]. Hierfür sind der Aufbau eines Vertrauensverhältnisses und transparente Schutz- und Sicherheitsmechanismen wichtig [13]. Im deutschen Kontext gilt es, für die Beurteilung der Zugangsvoraussetzungen die oben genannten Kriterien für Freiverantwortlichkeit zu prüfen. Allerdings sind diese für eine verlässliche praktische Anwendung aktuell teils unzureichend operationalisiert [18]. Auch hierzu sollte die genannte S2k-Leitlinie zukünftig Orientierung bieten [19]. Sofern die Gesundheitsfachperson diese Prüfung nicht selbst durchführt, kann es eine Aufgabe sein, bei der Vermittlung und Koordination der Beurteilungstermine zu unterstützten [12].

### Vorbereitung

Sofern die Erfüllung der Voraussetzungen festgestellt wurde, schließt sich die Vorbereitungsphase an. In dieser wird die Durchführung des assistierten Suizids konkret geplant [1]. Beispielsweise geht es darum, mit der sterbewilligen Person zu besprechen, wann, wo und in Anwesenheit welcher Personen der assistierte Suizid durchgeführt werden soll. Auch Informationen zum Ablauf der Durchführung und zu möglichen Komplikationen werden vermittelt, die emotionale Verarbeitung wird unterstützt sowie Familie und Freunde einbezogen [1, 13]. Die Planung unterscheidet sich je nach Versorgungssetting [12].

### Durchführung.

Für die Durchführung des assistierten Suizids werden Aufgaben benannt wie – sofern möglich – die Unterstützung der Umsetzung entsprechend den Vorstellungen der sterbewilligen Person, die erneute Überprüfung der Freiverantwortlichkeit und des fortbestehenden Wunsches nach assistiertem Suizid, ggf. die Unterstützung bei der Verabreichung des Medikaments (z. B. Legen eines Zugangs, Anmischen des Medikaments) sowie ggf. die Anwesenheit während des Sterbeprozesses [12, 13]. Auch wird hier betont, dass Pünktlichkeit, Verlässlichkeit und gute Organisation in dieser vulnerablen Situation essenziell sind [13]. Zudem ist die Betreuung der Angehörigen während der Durchführung relevant [13].

### Nachsorge.

Nachdem die sterbewillige Person verstorben ist, folgt die Nachsorge. Diese beinhaltet abhängig von den jeweiligen Vorgaben formale Aufgaben wie Dokumentationspflichten, Meldepflichten und das Ausfüllen des Totenscheins [12]. Zudem kann in dieser Phase die kurz- und gegebenenfalls längerfristige Betreuung der Angehörigen eine Aufgabe sein [13]. Auch ein Austausch oder eine strukturierte Nachbesprechung zur fachlichen und persönlichen Reflexion (Debriefing) mit Kolleg:innen kann zu den Aufgaben im Rahmen der Nachsorge gehören [12].

### Weitere mögliche Aufgaben.

Über die konkreten Aufgaben in der Versorgung der sterbewilligen Personen hinaus können die Unterstützung von Kolleg:innen z. B. im Rahmen von supervisorischen Angeboten sowie die Selbstfürsorge weitere Aufgaben sein [21, 22]. Zudem können sich Gesundheitsfachpersonen in mit assistiertem Suizid betrauten Gremien engagieren [22, 23].

## Wie *müssen* und *dürfen* sich Psychotherapeut:innen bei Anfragen nach assistiertem Suizid beteiligen?

Aufgrund der oben beschriebenen ausstehenden Neuregelung der Suizidassistenz in Deutschland bestehen derzeit keine spezifischen Pflichten für Psychotherapeut:innen im Kontext des assistierten Suizids [4]. Zwar sah einer der Gesetzesentwürfe vor, Psychotherapeut:innen bei der Beurteilung der Freiverantwortlichkeit und der Zumutbarkeit eines zweiten Untersuchungstermins Fachärzt:innen für Psychiatrie und Psychotherapie gleichzustellen [8], auch hieraus hätten sich jedoch aufgrund des Rechts auf Ablehnung einer Beteiligung aus Gewissensgründen keine verpflichtenden Aufgaben für Psychotherapeut:innen ergeben [3].

Insofern gelten für Psychotherapeut:innen bezüglich assistierten Suizids grundsätzlich dieselben Pflichten und Rechte wie für ihre Arbeit in anderen Bereichen. Die Musterberufsordnung der Bundespsychotherapeutenkammer legt als Ziele psychotherapeutischen Handelns fest, „Krankheiten vorzubeugen und zu heilen, Gesundheit zu fördern und zu erhalten sowie Leiden zu lindern“ [24, S. 4]. Ein analoger Satz zum 2024 aus der ärztlichen Musterberufsordnung gestrichenen Satz: „Sie [Ärztinnen und Ärzte] dürfen keine Hilfe zur Selbsttötung leisten“ [25, 26], findet sich in der psychotherapeutischen Musterberufsordnung nicht. Tätigkeitsfelder von Psychotherapeut:innen laut deren Musterberufsordnung seien neben der kurativen Behandlung unter anderem die palliative Versorgung sowie die Aus‑, Fort- und Weiterbildung und Forschung [24]. Wichtig sei, dass Psychotherapeut:innen ethische Prinzipien und die Würde der Patient:innen achten [24]. In den Sorgfaltspflichten wird sowohl eine diagnostische Einordnung als auch, wenn erforderlich, die Kooperation mit anderen Gesundheitsfachpersonen benannt [24]. Auch kann laut Musterberufsordnung das Verfassen von Gutachten eine Aufgabe von Psychotherapeut:innen sein, sofern sie über ausreichende Fachkenntnisse und Erfahrung verfügen und auf die Abgrenzung von begutachtender und unterstützend-therapeutischer Tätigkeit achten [24].

Assistierter Suizid oder verwandte Begriffe werden in der psychotherapeutischen Musterberufsordnung nicht explizit genannt. Die beschriebenen Inhalte erscheinen jedoch potenziell passend und hilfreich im Kontext von Anfragen nach assistiertem Suizid. Zum aktuellen Zeitpunkt konnten keine eindeutigen oder „harten“ rechtlichen Regelungen gefunden werden, die die Rechte und Pflichten von Psychotherapeut:innen in diesem Kontext festlegen.

## Wie *können* sich Psychotherapeut:innen bei Anfragen nach assistiertem Suizid beteiligen?

Neben dem, was Psychotherapeut:innen im rechtlichen Sinne müssen und dürfen, stellt sich die Frage, welche Fähigkeiten sie besitzen, die bei Anfragen nach assistiertem Suizid hilfreich sein können.

Sammlungen psychotherapeutischer Kompetenzen korrespondieren mit den in der Musterberufsordnung beschriebenen Inhalten. Beispielsweise wird im Psychotherapeutengesetz beschrieben, dass die psychotherapeutische Ausbildung unter anderem dazu befähigt, psychische Störungen festzustellen und zu behandeln bzw. an Dritte zu verweisen, Patient:innen sowie andere beteiligte Personen zu informieren und aufzuklären, gutachterlich tätig zu sein, interdisziplinär zusammenzuarbeiten und berufsethische Aspekte einzubeziehen ([27], § 7, Absatz 3). Auch die Bundespsychotherapeutenkammer hat sich zu psychotherapeutischen Kompetenzen positioniert, wobei die drei Bereiche „Faktenwissen“, „Handlungs- und Begründungswissen“ sowie „Handlungskompetenz und professionelle Haltung“ aufgegriffen wurden [28]. Neben den bereits benannten Aspekten werden in diesem Dokument die umfassende Befähigung von Psychotherapeut:innen zu sowohl gesunden als auch pathologischen psychischen Funktionen und Prozessen als auch zum Umgang mit komplexen und mehrdeutigen Sachverhalten deutlich. Zudem findet man ähnliche internationale Kompetenzzusammenstellungen, wie beispielsweise von der European Association of Psychotherapy [29]. In deren Dokument werden unter anderem die Anforderungen und Fähigkeiten hinsichtlich Selbstreflexion und persönlicher Weiterentwicklung sowie Beziehungsgestaltung betont. Zudem werden das Nutzen von Supervision und Selbstfürsorge als wichtige Kompetenzen benannt.

Setzt man die zuvor beschriebenen Aufgaben bei Anfragen nach assistiertem Suizid mit den benannten Pflichten und Kompetenzen von Psychotherapeut:innen in Verbindung, wird deutlich, dass Psychotherapeut:innen über einschlägige Expertise verfügen, die sie sehr gut für die Übernahme von einigen Aufgaben bei Anfragen nach assistiertem Suizid qualifizieren. Hinsichtlich der Querschnittsaufgaben sowie der Aufgaben in der Anfangs- und Abwägungsphase sind Psychotherapeut:innen aufgrund ihrer Kommunikationsfähigkeiten auch in komplexen und mehrdeutigen Sachverhalten, ihrer Expertise im Beziehungsaufbau und ihrer Kompetenzen in der interdisziplinären Zusammenarbeit und Vernetzung sehr gut qualifiziert.

In verschiedenen Phasen wie der Anfangs- und Abwägungs- und der Vorbereitungsphase könnten Psychotherapeut:innen dabei unterstützen, herauszufinden, was die Bedürfnisse und Werte einer Person sind. Psychotherapeutische Arbeit ist durch eine starke Personenzentrierung und ein Zurückstellen eigener Werturteile geprägt, was bei Anfragen nach assistiertem Suizid essenziell ist. Konkret könn(t)en Psychotherapeut:innen beispielsweise „Motivational Interviewing“ nutzen, um Personen mit einem Wunsch nach assistiertem Suizid bei der Klärung ihrer Motivation zu unterstützen. Motivational Interviewing ist ein ursprünglich für die Suchtbehandlung entwickelter Ansatz, der mittlerweile in vielen Bereichen Anwendung findet und die Klärung von motivationalen Unklarheiten und Ambivalenzen unterstützen kann [30]. Zudem sind assistierte Suizide von Personen mit psychischen Störungen grundsätzlich nicht ausgeschlossen, sofern die psychische Störung die Freiverantwortlichkeit nicht einschränkt. In solchen Fällen könn(t)en Psychotherapeut:innen über Behandlungsoptionen der psychischen Störung als mögliche Alternative zum assistierten Suizid aufklären und so eine informierte Entscheidung für oder gegen einen assistierten Suizid unterstützen.

Für die Beurteilungsphase, welche in Deutschland aktuell primär aus der Begutachtung der Freiverantwortlichkeit besteht, weisen Psychotherapeut:innen ebenfalls wichtige Kenntnisse, beispielweise in psychologischer Diagnostik, auf. Psychotherapeut:innen sind qualifiziert, differenziert zu beurteilen, ob und inwieweit Wünsche nach assistiertem Suizid Ausdruck fehlender oder anzuzweifelnder Freiverantwortlichkeit sind oder als freiverantwortliche Entscheidung verstanden werden können. Für fachlich fundierte Beurteilung bedarf es jedoch – wie bereits erwähnt – einer übergeordneten Klärung der Operationalisierung und Erhebung der Kriterien der Freiverantwortlichkeit [18, 19].

Weniger relevant erscheint die psychotherapeutische Expertise bei der Durchführung assistierter Suizide. Bei der Unterstützung von Angehörigen und Kolleg:innen unter anderem in der Nachsorgephase könnten sich psychotherapeutische Kenntnisse wiederum gut nutzen lassen. Die Begleitung von Angehörigen ist eine psychotherapeutische Kernkompetenz. Diese Aufgabe übernehmen Psychotherapeut:innen beispielsweise bereits im Rahmen der Spezialambulanz zur Suizidprävention der Medical School Berlin, welche ihr Angebot explizit auch an Hinterbliebene nach assistiertem Suizid richtet [31].

Insgesamt weisen die Kompetenzen von Psychotherapeut:innen eine hohe Relevanz und Passung im Kontext von Anfragen nach assistiertem Suizid auf. Gleichwohl stellt sich die Frage, ob und wie sich die Kompetenzen auf diesen Kontext ausweiten lassen. Ein relevanter Unterschied zu vielen anderen psychotherapeutischen Tätigkeitsfeldern besteht darin, dass Anfragen nach assistiertem Suizid die Beendigung des eigenen Lebens als explizites Ziel haben, welches potenziell auch von dem Psychotherapeuten/der Psychotherapeutin mitgetragen würde. Unter den psychotherapeutischen Tätigkeitsfeldern finden sich zwar solche, in denen potenziell über Leben und Sterben einer Person mitentscheiden wird. Dies ist beispielsweise in der Transplantationspsychologie der Fall, wenn Psychotherapeut:innen an der Begutachtung beteiligt sind, ob eine Person ein Organ erhalten kann [32]. In diesen Kontexten ist das selbstbestimmte Sterben jedoch weder explizites Ziel noch Anliegen. Welche Unterschiede sich hierdurch für die psychotherapeutische Rolle und Arbeit ergeben und welche Konsequenzen dies hat, bedarf einer sorgfältigen Reflexion sowie der Entwicklung entsprechender sprachlicher und professioneller Handlungssicherheit.

## Wie *sollten* sich Psychotherapeut:innen bei Anfragen nach assistiertem Suizid beteiligen?

Die normative Frage, ob und wie sich Psychotherapeut:innen bei Anfragen nach assistiertem Suizid beteiligen sollten, kann aus verschiedenen Perspektiven betrachtet werden. In diesem Abschnitt werden Abwägungen auf der theoretischen und der empirischen Ebene vorgenommen, ohne den Anspruch auf Vollständigkeit oder abschließende Antworten. Alle Abwägungen weisen Relevanz für Psychotherapeut:innen auf, wobei einige auch auf andere Berufsgruppen anwendbar sind.

Als Erstes stellt sich die Frage, ob die einschlägige Expertise von Psychotherapeut:innen impliziert, dass sie sich auch beteiligen sollten. Zugespitzt formuliert: Die Fähigkeit, etwas zu tun, begründet für sich genommen noch keine normative Verpflichtung, es auch zu tun. Ein mögliches Gegenargument bzw. ein potenzieller Nachteil der Beteiligung besteht in einer Vermischung von Unterstützungs- und Beurteilungsfunktion, worauf auch die psychotherapeutische Musterberufsordnung hinweist [24]. Eine solche Vermischung könnte sich beispielsweise negativ auf das Vertrauensverhältnis und den „sicheren Raum“, den psychotherapeutische Gespräche darstellen und in dem alles thematisiert werden kann, auswirken.

Die Sorge hinsichtlich einer Vermischung von Unterstützungs- und Beurteilungsfunktion wurde auch in einer qualitativ-empirischen Befragung von Psychoonkolog:innen zu deren aktueller und möglicher zukünftiger Rolle bei Anfragen nach assistiertem Suizid identifiziert [33]. In dieser Befragung sahen sich die Psychoonkolog:innen primär in einer kommunikativ-unterstützenden Rolle und nur manche konnten sich auch eine evaluative Rolle vorstellen. Auch für Ärzt:innen wurden im Kontext der aktuellen gesetzlichen Änderungen in Großbritannien ähnliche Bedenken hinsichtlich der verschiedenen Funktionen geäußert [34]. Eine Aufteilung der Aufgaben auf verschiedene Personen und somit die Trennung der Funktionen könnte dieser Schwierigkeit begegnen. Moralischer Stress aufseiten der Psychotherapeut:innen stellt ein weiteres potenzielles Risiko einer Beteiligung an Anfragen nach assistiertem Suizid dar. Zugleich sind Psychotherapeut:innen durch ihre Ausbildung, Supervision und Intervision darauf vorbereitet, selbstfürsorglich zu handeln und mit moralischem Stress umzugehen.

Für eine Beteiligung von Psychotherapeut:innen an Anfragen nach assistiertem Suizid wurde sich neben der genannten qualitativ-empirischen Befragung [33] auch in einer internationalen Übersichtsarbeit [35] und einer quantitativen Befragung in Portugal und Luxemburg [36] ausgesprochen. In der Übersichtsarbeit wurden die folgenden Hauptaufgabenfelder identifiziert: Evaluation der Entscheidungsfähigkeit, Kommunikation, psychologische Beratung, Forschung, Aus- und Fortbildung und politisches Engagement [35]. Die Vorteile einer Beteiligung von Psychotherapeut:innen an Anfragen nach assistiertem Suizid aufgrund ihrer einschlägigen Kompetenzen scheinen die möglichen Nachteile durch die Erweiterung des psychotherapeutischen Rollen- und Funktionsspektrums zu überwiegen.

Eine klare Grenze des Sich-beteiligen-Sollens auf individueller Ebene stellt jedoch das vom Bundesverfassungsgericht postulierte Recht auf Ablehnung der Beteiligung aus Gewissensgründen dar. Auch Psychotherapeut:innen haben entsprechend das Recht, frei zu entscheiden, ob sie sich persönlich an Anfragen nach assistiertem Suizid beteiligen möchten. Dieser Bedarf wurde auch in der qualitativen Interviewstudie mit Psychoonkolog:innen betont [33]. Gleichzeitig ist in Deutschland bislang offen, ob eine Ablehnung der Beteiligung aus Gewissensgründen bedeuten würde, dass man verpflichtet ist, die anfragende Person an eine:n andere:n geeignete:n Behandler:in zu verweisen. In einigen Ländern besteht diese Verpflichtung [20]. Auf kollektiver Ebene wird hierbei gewissermaßen dem individuellen Recht auf Ablehnung einer Beteiligung das Recht auf assistierten Suizid gegenübergestellt.

## Fazit

Zusammenfassend ergibt sich aus den einschlägigen Kompetenzen von Psychotherapeut:innen potenziell ein großer Nutzen durch deren Beteiligung an Anfragen nach assistiertem Suizid, weshalb diese zu empfehlen ist. Gleichzeitig muss jede Beteiligung auf Freiwilligkeit beruhen und auf eine klare Abgrenzung von Unterstützungs- und begutachtenden Aufgaben geachtet werden. Die potenziellen Konsequenzen einer psychotherapeutischen Beteiligung an Anfragen nach assistiertem Suizid für die psychotherapeutische Arbeit und das Bild der Berufsgruppe in der Gesellschaft sollten sorgfältig reflektiert und proaktiv kommuniziert werden.
